# Vitamin D and Curcumin Mitigate ACLT + MMx-Induced Knee Osteoarthritis by Improving Antioxidant-Related Nrf2/HO-1 Expression and Reducing Synovial Inflammation

**DOI:** 10.3390/nu18142288

**Published:** 2026-07-13

**Authors:** Lokesh Kumar Mende, Yaswanth Kuthati, Chih-Shung Wong

**Affiliations:** 1 Department of Anesthesiology, Cathay General Hospital, Taipei 280, Taiwan; 2 Graduate Institute of Medical Sciences, National Defense Medical University, Taipei 280, Taiwan

**Keywords:** knee osteoarthritis, chondroprotection, oxidative stress, vitamin D, curcumin, pain, inflammation, synovial membrane, Nrf2/HO-1 expression

## Abstract

**Background**: Osteoarthritis (OA) is mainly driven by mechanical damage together with persistent oxidative stress and synovial inflammation, leading to progressive cartilage loss and functional impairment. We evaluated the protective effect of vitamin D and curcumin alone or in combination on OA progression and outcomes in an anterior cruciate ligament transection plus medial meniscectomy (ACLT + MMx) rat model. **Methods**: OA was induced by ACLT + MMx surgery. Rats were assigned to Sham, OA-control (Control), vitamin D, curcumin, and Vitamin D + Curcumin groups (*n* = 6/group). Functional outcomes (knee width/swelling and weight-bearing Δ force) and body weight were monitored for 12 weeks. Cartilage damage was assessed by H&E histology and quantitative histomorphometry (cartilage thickness/width) together with degeneration OARSI score assessment. Mechanistic readouts included qPCR for Nrf2 and HO-1, serum oxidative stress markers (MDA, GSSG, CAT), and synovial cytokines (TNF-α, IL-1β, IL-10, MCP-1). **Results**: OA-control rats showed clear functional deterioration (greater knee swelling and impaired weight bearing), pronounced histological cartilage degeneration, and higher degeneration/OARSI scores. Vitamin D or curcumin treatment improved functional measures, preserved cartilage structure, and reduced cartilage degeneration indices. These benefits were generally strongest with the combined Vitamin D + Curcumin treatment, which showed the greatest overall preservation of cartilage integrity. Treatments were associated with increased synovial Nrf2/HO-1 mRNA expression, improved oxidative stress balance, and a shift toward a less inflammatory synovial cytokine profile. **Conclusions**: Vitamin D and curcumin, particularly in combination, showed cartilage-protective and functional benefits in ACLT + MMx-induced OA, possibly through coordinated reduction in oxidative stress and synovial inflammation together with increased antioxidant-related Nrf2/HO-1 expression.

## 1. Introduction

Osteoarthritis (OA) is a whole-joint disorder rather than a disease restricted to articular cartilage. Cartilage, synovium, subchondral bone, ligaments, menisci, and periarticular tissues all contribute to its clinical and structural progression. Current concepts describe OA as an active biological process in which abnormal mechanical loading, tissue stress, low-grade inflammation, and an insufficient repair response gradually disturb joint homeostasis [1,2,3,4]. In the knee joint, these pathological changes gradually disturb cartilage homeostasis, reduce extracellular matrix integrity, and finally result in cartilage erosion, joint stiffness, pain, swelling, and functional disability [1,2]. Post-traumatic OA is commonly associated with joint instability and abnormal mechanical loading. Experimental models such as anterior cruciate ligament transection combined with medial meniscectomy (ACLT + MMx) are widely used because they reproduce important features of progressive knee OA, including cartilage surface damage, matrix loss, synovial activation, and weight-bearing imbalance [5,6,7]. In preclinical studies, these structural changes are commonly evaluated using H&E staining, cartilage morphometry, degeneration scoring, and Osteoarthritis Research Society International (OARSI)-based histological scoring systems [8,9]. Such assessments provide a reliable way to compare cartilage damage severity and treatment-related chondroprotection across experimental groups. Oxidative stress is a key contributor to OA progression. Under normal conditions, chondrocytes maintain a balance between reactive oxygen species generation and antioxidant defense. However, during OA, excessive oxidative stress can impair mitochondrial function, promote chondrocyte apoptosis or senescence, reduce type II collagen and aggrecan synthesis, and increase cartilage-degrading enzymes such as matrix metalloproteinases [10,11,12]. Oxidative stress also interacts closely with inflammation, creating a harmful cycle in which inflammatory cytokines increase oxidative damage, while oxidative stress further activates catabolic and inflammatory pathways in cartilage and synovial tissues [10,13].

Synovial inflammation is another important driver of OA severity. Activated synovial cells and infiltrating immune cells release cytokines and chemokines, including IL-6, TNF-α, IL-1β, and MCP-1, which can promote cartilage matrix degradation, pain sensitization, and further recruitment of inflammatory cells [14,15,16,17]. In contrast, anti-inflammatory mediators such as IL-10 may help counterbalance excessive inflammatory signaling and support a more protective joint microenvironment [18]. Therefore, targeting both oxidative stress and synovial inflammation may provide better disease control than focusing on cartilage structure alone. The nuclear factor erythroid 2-related factor 2/heme oxygenase-1 pathway, known as the Nrf2/HO-1 pathway, is one of the major endogenous antioxidant defense systems. Activation of Nrf2 promotes the transcription of antioxidant and cytoprotective genes, including HO-1, which helps reduce oxidative injury and regulate inflammatory responses in joint tissues [19,20]. In OA-related studies, Nrf2 activation has been associated with improved chondrocyte survival, reduced apoptosis, lower oxidative damage, and better maintenance of cartilage matrix homeostasis [19,20,21]. Thus, the Nrf2/HO-1 pathway represents a biologically relevant therapeutic target for slowing OA-associated cartilage degeneration.

Vitamin D has biological roles beyond calcium and bone metabolism. It may influence cartilage integrity, chondrocyte activity, inflammatory responses, and subchondral bone remodeling. Experimental evidence suggests that vitamin D supplementation can reduce OA-associated pain, cartilage destruction, inflammatory cytokine expression, and matrix metalloproteinase activity [22,23]. Clinical studies have also linked vitamin D deficiency with oxidative stress and increased MMP activity in knee OA; however, supplementation responses may depend on baseline vitamin D status, disease stage, and patient heterogeneity [24]. Curcumin, the major polyphenolic compound of Curcuma longa, has attracted attention because of its antioxidant, anti-inflammatory, and cartilage-protective properties. Previous studies have reported that curcumin can reduce inflammatory signaling, suppress oxidative stress, protect chondrocytes, and improve OA-related pain and joint function [25,26,27,28]. Mechanistically, curcumin-mediated chondroprotection has been linked to activation of the Nrf2/ARE pathway, inhibition of catabolic responses, and improvement of mitochondrial quality-control pathways such as mitophagy [25,29]. These findings support curcumin as a promising natural compound for targeting multiple OA-related pathways. However, the clinical translation of curcumin remains challenging because of its low aqueous solubility, poor oral bioavailability, rapid metabolism, and limited systemic absorption. Therefore, the differences between preclinical dosing in rats and practical human use should be considered when interpreting the therapeutic potential of curcumin in OA.

Because OA progression involves several interconnected mechanisms, combination therapy may offer broader protection than single-agent treatment. Vitamin D may support cartilage and bone-related homeostasis while reducing inflammation, whereas curcumin may strongly regulate oxidative stress, inflammatory signaling, and chondrocyte survival. Therefore, the present study investigated whether vitamin D and curcumin, alone or in combination, could protect against ACLT + MMx-induced knee OA in rats. The novelty has been clarified as the evaluation of combined vitamin D and curcumin treatment in this preclinical OA model, with integrated assessment of functional outcomes, cartilage histology, Nrf2/HO-1 antioxidant signaling, oxidative stress markers, and synovial inflammatory cytokines.

## 2. Materials and Methods

### 2.1. Experimental Design

**Figure 1:**
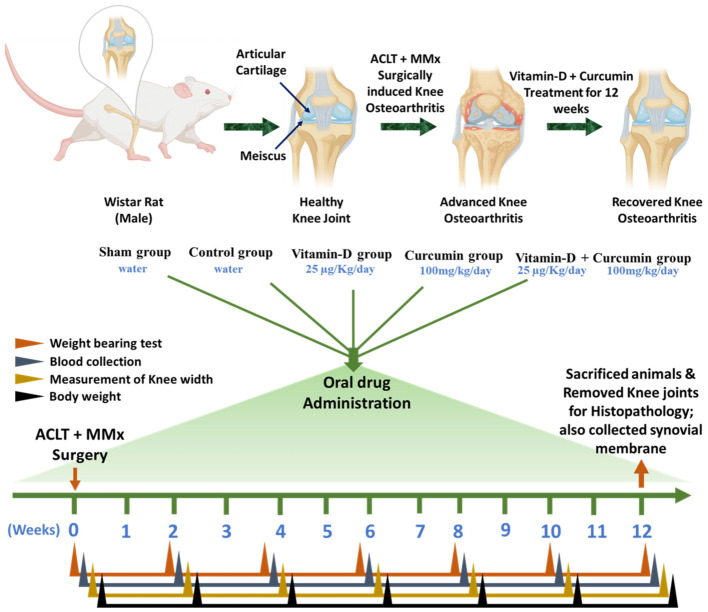
Experimental workflow and time frame.

The study was designed to determine whether vitamin D, curcumin, or their combined administration could reduce ACLT + MMx-induced OA progression in vivo. The overall experimental workflow and time frame are shown in Figure 1. Rats were distributed into five groups: Sham, OA-control (Control), vitamin D (25 µg/kg/day), curcumin (100 mg/kg/day), and Vitamin D + Curcumin (25 µg/kg/day + 100 mg/kg/day). Treatments were delivered orally once daily for 12 weeks. Body weight, knee width, static weight-bearing distribution, and blood sampling were performed at baseline and at weeks 2, 4, 6, 8, 10, and 12. At the end of the experiment, oa were euthanized and knee joints and synovial membranes were collected for histological, molecular, oxidative stress, and cytokine assessments.

### 2.2. ACLT + MMx-Induced Knee OA Model

The OA procedure followed previously described approaches with minor experimental adaptation [30,31]. Male Wistar rats weighing 330–350 g (10 to 12 weeks old) were anesthetized with isoflurane. After shaving and povidone–iodine disinfection, ACLT + MMx surgery was performed on the right knee. A medial capsular incision was made to expose the joint, the anterior cruciate ligament was transected, and the medial meniscus was removed using fine surgical scissors. The joint capsule and skin were then closed with appropriate sutures. To reduce postoperative infection risk, the wound was disinfected and cefazolin (100 mg/kg/day) was administered intramuscularly for three days. Sham-operated rats underwent the same skin incision and joint exposure without ligament transection or meniscal removal.

### 2.3. Drug Administration and Grouping

Thirty rats were assigned to five experimental groups using a simple random allocation procedure: Sham (water), OA-control (water), vitamin D, curcumin, and Vitamin D + Curcumin groups, with 6 rats in each group. The number of animals in each group was selected based on commonly used group sizes in previous ACLT + MMx-induced osteoarthritis studies and our prior experience with this experimental model. No formal a priori sample size estimation or power calculation was performed before the experiment. Therefore, the present study should be regarded as a preclinical exploratory study designed to evaluate treatment-related effects while following the ethical principle of reducing animal use. Animals were excluded only if severe postoperative complications, infection, incomplete surgical induction, abnormal baseline measurements, or technical failure during tissue collection or biochemical analysis occurred. Vitamin D and curcumin were prepared for oral delivery. The vitamin D group received (25 µg/kg/day), the curcumin group received (100 mg/kg/day), and the combined-treatment group received both vitamin D (25 µg/kg/day) and curcumin (100 mg/kg/day). Treatments were administered daily for 12 weeks.

### 2.4. Knee Width and Weight-Bearing Assessment

Knee width was measured every two weeks using a caliper, and the contralateral knee served as the internal non-operated reference. Static weight-bearing distribution was evaluated at the same time points using an in-capacitance-based weight-bearing system. Rats were placed with both hind paws on the sensor plates in an inclined chamber, and the force applied by each hind limb was recorded after calibration. Weight-bearing ∆ force was calculated as the difference between the non-operated and operated limbs. Higher ∆ force values indicate reduced loading on the injured side and are interpreted as a functional sign of OA-related discomfort.

### 2.5. Histopathology Examination of Knee Joints

At week 12, animals were euthanized and knee joints were collected. Samples were fixed in 10% formalin for 3 days, decalcified in 12.5% EDTA disodium solution (pH 7.0) for 4 weeks, embedded in paraffin, and sectioned at 5 um thickness. Hematoxylin and eosin (H&E) staining was then performed to assess cartilage morphology, matrix integrity, chondrocyte organization, and osteoarthritis-related histopathological changes. Stained sections were examined for cartilage surface integrity, matrix preservation, chondrocyte organization, cartilage thickness, and degeneration severity. Images were captured using a ZEISS imaging system (Oberkochen, Germany), and cartilage damage was evaluated using modified OARSI-based histological criteria. Cartilage matrix loss, degeneration score, total degeneration width, significant degeneration width, and zonal depth ratio were quantified for comparison among groups.

### 2.6. Collection and Storage of Synovial Membrane

Synovial membrane tissues were collected from the knee joints after euthanasia. Briefly, the knee joint was excised with small femoral and tibial fragments to preserve the joint structure during dissection. The joint cavity was opened from the femoral side, and the patella, patellar ligament, and synovium-covered joint capsule were exposed. Under a dissecting microscope, the synovial membrane was carefully separated from the patella, patellar ligament, and surrounding capsule using fine forceps and scissors. Contamination with cartilage, meniscus, bone, muscle, or excess fat was avoided. Collected tissue was briefly rinsed in ice-cold PBS, blotted dry, snap-frozen in liquid nitrogen, and stored at −80 °C until qPCR, protein, or cytokine analysis [32].

### 2.7. Quantitative Real-Time PCR

Total RNA was extracted from frozen synovial tissues using RNAiso Plus (Takara Bio Inc., Kusatsu, Shiga, Japan) according to the manufacturer’s instructions. cDNA was synthesized using the PrimeScript RT reagent kit with gDNA Eraser (Takara Bio Inc.). Quantitative PCR was performed using a Bio-Rad iCycler system and TB Green Premix Ex Taq II (Takara Bio Inc.). The cycling conditions included denaturation at 95 °C for 5 s and annealing/extension at 60 °C for 30 s. Relative Nrf2 and HO-1 mRNA expression was calculated against the control reference and reported as mean ± SD [33].

### 2.8. Measurement of Synovial Cytokines

At week 12, after euthanasia, frozen synovial membrane tissues were homogenized in ice-cold PBS or lysis buffer supplemented with protease inhibitors. After centrifugation, supernatants were collected for cytokine measurements. IL-10, TNF-α, IL-1β, and MCP-1 concentrations were determined using colorimetric ELISA kits (Calbiochem-Novabiochem Co., Milan, Italy) following the manufacturer instructions [34].

### 2.9. Serum Oxidative Stress Markers

Before euthanasia and at the scheduled time points, blood samples were collected from the tail vein. Samples were centrifuged at 3000× *g* for 15 min, and serum was stored at −80 °C until analysis. MDA, GSSG, and CAT were measured using commercial kits from Abcam Co., Ltd. (Cambridge, UK), according to the manufacturer instructions and established protocols [35]. Measurements were performed in triplicate.

### 2.10. Statistical Analysis

Data are presented as mean ± SD. Graphs were generated using GraphPad Prism version 9. Group comparisons were performed using one-way analysis of variance followed by Tukey’s post hoc multiple-comparison test. A *p* value < 0.05 was considered statistically significant. Significance levels were expressed as * *p* < 0.05, ** *p* < 0.01, and *** *p* < 0.001.

## 3. Results

This study demonstrated that vitamin D and curcumin, particularly in combination, effectively reduced pain and inflammation in ACLT + MMx-induced knee OA in rats. The ACLT + MMx procedure successfully induced knee OA-like changes in rats, and synovial membrane tissue was reliably collected for cytokine and molecular analyses. Body weight increased gradually from week 0 to week 12 in all groups, with no obvious weight loss, indicating that the surgical procedure and oral administration of vitamin D, curcumin, or their combination were generally well tolerated. Compared with OA-control rats, animals treated with vitamin D, curcumin, and especially Vitamin D + Curcumin showed improved weight-bearing distribution and reduced knee swelling. Histological evaluation showed marked cartilage degeneration in OA-control rats, including cartilage thinning, matrix disruption, chondrocyte loss, and increased cartilage degradation and OARSI scores. These changes were reduced by vitamin D and curcumin treatment, with the combined treatment showing the greatest cartilage preservation. In addition, vitamin D and curcumin increased synovial Nrf2 and HO-1 mRNA expression, reduced serum MDA and GSSG levels, improved CAT activity, decreased synovial TNF-α, IL-1β, and MCP-1 levels, and increased IL-10 levels. Overall, the combined treatment produced stronger improvements than either single treatment alone.

### 3.1. Knee Dissection, Synovial Tissue Collection, and Body Weight Changes in ACLT + MMx-Induced OA

**Figure 2:**
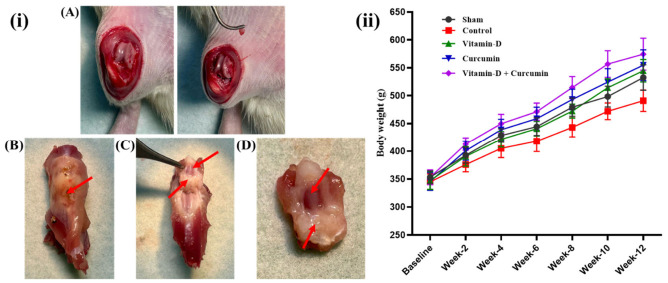
(**i**) **Knee dissection**: (**A**) Representative photograph of the knee joint after ACLT + MMx surgery-induced osteoarthritis. (**B**) Excise knee with small femur/tibia fragments; open from the femoral side. (**C**) Expose patella and patellar ligament under synovium. (**D**) Patella with synovium resting on the ligament. Red arrows indicate the synovium-covered region around the patella and patellar ligament used for synovial membrane identification and collection. (**ii**) **Body weight**: Measured in Sham, OA-control (Control), Vitamin D, Curcumin, and Vitamin D + Curcumin (*n* = 6) at weeks 0, 2, 4, 6, 8, 10, and 12.

**Figure 3:**
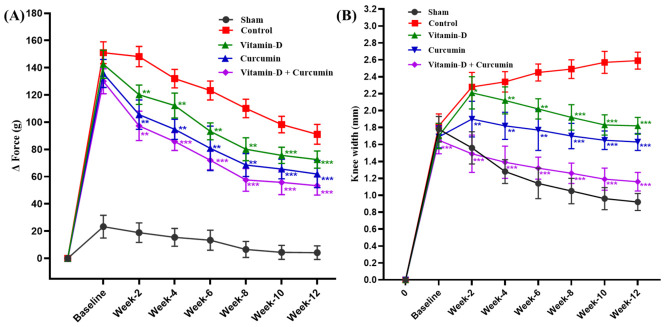
(**A**) weight-bearing (Δ force) tracked in Sham, OA-control (Control), Vitamin D, Curcumin, and Vitamin D + Curcumin rats (*n* = 6/group) at weeks 0, 2, 4, 6, 8, 10, and 12. (**B**) Knee width (mm). Values are mean ± SD; significance shown as * *p* < 0.05, ** *p* < 0.01, *** *p* < 0.001.

The ACLT + MMx-induced knee OA model was established in the right knee, and the synovial membrane was clearly exposed during dissection (Figure 2i). The knee joint was excised with small femoral and tibial fragments and opened from the femoral side to identify the patella, patellar ligament, and synovium-covered region for subsequent cytokine and molecular analyses. Body weight increased steadily from week 0 to week 12 in all groups, with no apparent treatment-related weight loss (Figure 2ii). This finding indicated that ACLT + MMx surgery and oral treatment with vitamin D, curcumin, or Vitamin D + Curcumin was well tolerated during the experimental period. Compared with OA-control rats, vitamin D, curcumin, and Vitamin D + Curcumin-treated rats showed a significant reduction in weight-bearing Δ force (Figure 3A). Knee width was also lower in the treatment groups than in the OA-control group, with the greatest reduction observed in the combined treatment group (Figure 3B).

### 3.2. Vitamin D and Curcumin Attenuate Cartilage Injury in ACLT + MMx-Induced OA

**Figure 4:**
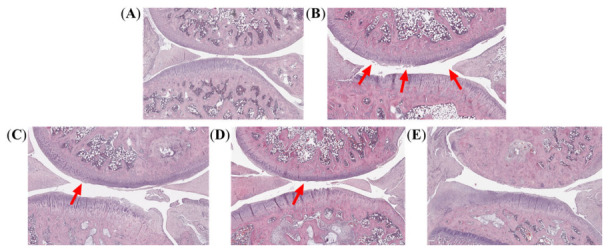
Representative knee joint histology. H&E-stained sections showing articular cartilage morphology in (**A**) Sham, (**B**) OA-control (Control), (**C**) Vitamin D, (**D**) Curcumin, and (**E**) Vitamin D + Curcumin groups (*n* = 6). Red arrows indicate representative OA-related cartilage lesions, including surface irregularity, cartilage thinning, matrix disruption, and chondrocyte loss. Scale bar = 250 µM.

**Figure 5:**
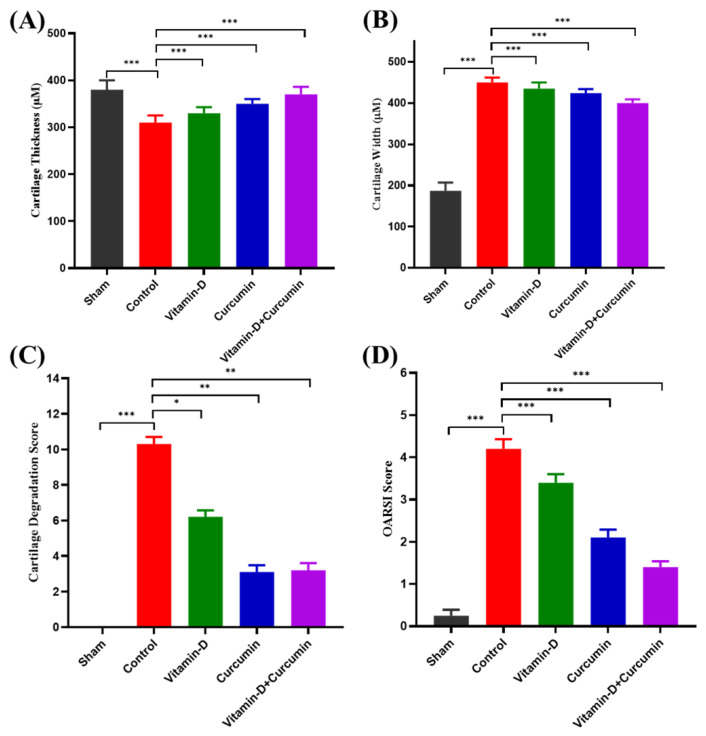
Cartilage histomorphometric analysis showing (**A**) Cartilage Thickness, (**B**) Cartilage width, (**C**) Cartilage degradation score, and (**D**) OARSI score in Sham, OA-control (Control), Vitamin D-treated, Curcumin-treated, and Vitamin D + Curcumin co-treated groups. Results are expressed as mean ± SD (*n* = 6 per group), with statistical significance indicated as * *p* < 0.05, ** *p* < 0.01, and *** *p* < 0.001.

Histological analysis showed clear differences in cartilage morphology among the experimental groups. Sham joints maintained a smooth articular surface, preserved matrix, and organized chondrocyte distribution (Figure 4A). In contrast, OA-control rats showed typical OA-related cartilage damage, including surface irregularity, cartilage thinning, chondrocyte loss, and extracellular matrix disruption (Figure 4B). This observation supports our earlier findings that vitamin D may alleviate OA-related pain and limit cartilage degeneration, partly by downregulating matrix metalloproteinase activity [23]. Treatment with vitamin D or curcumin reduced cartilage degeneration relative to the OA-control group, while the Vitamin D + Curcumin group showed the most evident preservation of cartilage structure (Figure 4C–E). These histological findings were supported by OARSI scoring and cartilage damage assessment shown in Table 1. Quantitative analysis further showed that ACLT + MMx surgery decreased cartilage thickness and increased cartilage width, cartilage degradation score, and OARSI score compared with the Sham group (Figure 5A–D). Vitamin D and curcumin improved these cartilage parameters. The combined treatment showed clear cartilage-preserving effects; however, some structural scoring outcomes, including cartilage degeneration and OARSI-related scores, were similar between the curcumin and Vitamin D + Curcumin groups. Quantitatively, cartilage thickness was reduced in the OA-control group (310 ± 15 µm) compared with the Sham group (380 ± 20 µm). Treatment improved cartilage thickness in the vitamin D group (330 ± 13 µm), curcumin group (350 ± 10 µm), and Vitamin D + Curcumin group (370 ± 16 µm). Similarly, the OARSI score was highest in OA-control rats (4.20 ± 0.23) and was reduced after vitamin D (3.40 ± 0.20), curcumin (2.10 ± 0.19), and combined treatment (1.40 ± 0.14).

### 3.3. Vitamin D and Curcumin Are Associated with Increased Synovial Nrf2/HO-1 mRNA Expression

**Figure 6:**
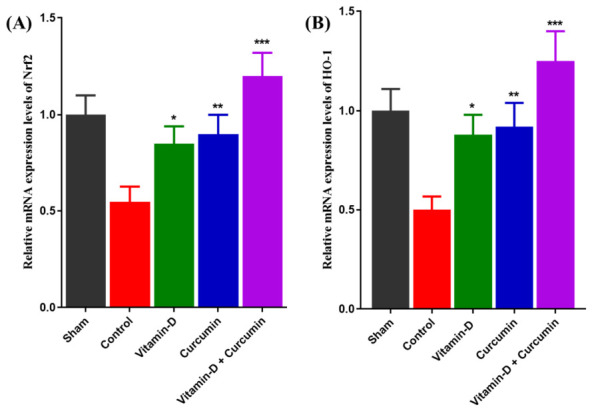
qPCR was used to quantify (**A**) Nrf2 and (**B**) HO-1 expression. Asterisks indicate statistically significant comparisons between Sham, OA-control (Control), Vitamin D-treated, Curcumin-treated, and Vitamin D + Curcumin co-treated groups. Results are expressed as mean ± SD (*n* = 6 per group), with statistical significance indicated as * *p* < 0.05, ** *p* < 0.01, and *** *p* < 0.001.

Nrf2 and HO-1 mRNA expression was markedly reduced in the OA-control group than in the Sham group (Figure 6A,B). Administration of either vitamin D or curcumin significantly upregulated both markers relative to the OA-control group. The Vitamin D + Curcumin group showed the highest Nrf2 and HO-1 expression among the treatment groups. Compared with the OA-control group, Nrf2 mRNA expression increased by approximately (0.85) in the vitamin D group, (0.90) in the curcumin group, and (1.2) in the Vitamin D + Curcumin group. HO-1 mRNA expression showed a similar pattern, with the greatest increase observed in the combined treatment group (1.25). These results suggest that vitamin D and curcumin co-treatment was associated with increased antioxidant-related Nrf2 and HO-1 mRNA expression in synovial tissue, but these mRNA-level findings should not be interpreted as direct proof of pathway activation [20].

### 3.4. Vitamin D and Curcumin Improve Serum Oxidative Stress Markers

**Figure 7:**
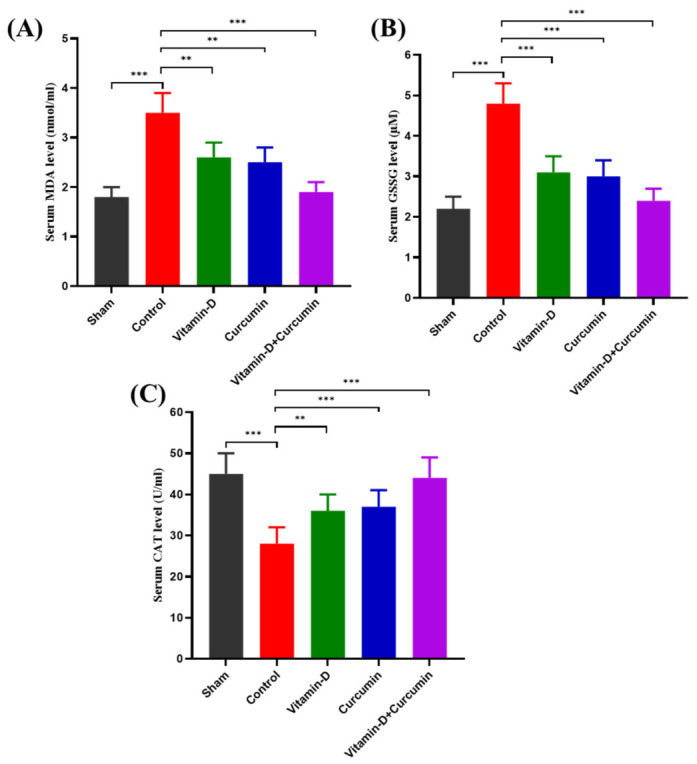
Serum oxidative stress over time (**A**) MDA, (**B**) GSSG, and (**C**) CAT tracked at weeks 0, 2, 4, 6, 8, 10, and 12 in Sham, OA-control (Control), Vitamin D-treated, Curcumin-treated, and Vitamin D + Curcumin co-treated groups. Results are expressed as mean ± SD (*n* = 6 per group), with statistical significance indicated as ** *p* < 0.01, and *** *p* < 0.001.

Serum oxidative stress markers were altered after ACLT + MMx surgery. OA-control rats showed higher MDA and GSSG levels and lower CAT activity compared with the Sham group (Figure 7A–C). Treatment with vitamin D or curcumin reduced MDA and GSSG levels and increased CAT activity compared with OA-control rats. The Vitamin D + Curcumin group showed the greatest improvement, with the lowest MDA and GSSG levels and the highest CAT activity among the treated groups [12]. At week 12, MDA was elevated in the OA-control group (3.50 ± 0.40) compared with the Sham group (1.80 ± 0.20) and was reduced after vitamin D (2.60 ± 0.30), curcumin (2.50 ± 0.30), and Vitamin D + Curcumin treatment (1.90 ± 0.20). GSSG showed a similar reduction after treatment, while CAT activity increased most clearly in the Vitamin D + Curcumin group (44.0 ± 5.0) compared with OA-control rats (28.0 ± 4.0).

### 3.5. Vitamin D and Curcumin Attenuate Synovial Inflammation

**Figure 8:**
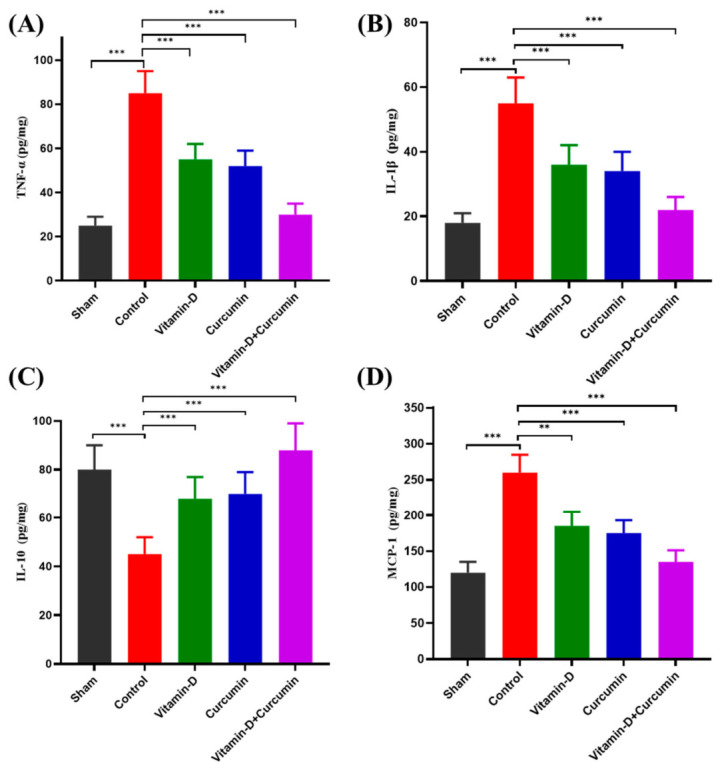
Synovial cytokine levels at the terminal endpoint in ACLT + MMx-induced knee OA rats. Synovial membrane homogenates collected at week 12 were used to measure (**A**) TNF-α, (**B**) IL-1β, (**C**) IL-10, and (**D**) MCP-1 in Sham, OA-control (Control), Vitamin D-treated, Curcumin-treated, and Vitamin D + Curcumin co-treated groups. These cytokine data represent a single endpoint measurement and not a longitudinal time-course assessment. Results are expressed as mean ± SD (*n* = 6 per group), with statistical significance indicated as ** *p* < 0.01, and *** *p* < 0.001.

Synovial cytokine analysis showed that ACLT + MMx surgery increased inflammatory cytokine levels in OA-control rats. TNF-α, IL-1β, and MCP-1 levels were higher in the OA-control group than in the Sham group, whereas IL-10 levels were reduced (Figure 8A–D). Treatment with vitamin D or curcumin decreased TNF-α, IL-1β, and MCP-1 levels and increased IL-10 levels compared with OA-control rats. The Vitamin D + Curcumin group showed the strongest cytokine modulation, with the lowest pro-inflammatory cytokine levels and the highest IL-10 level among the treated groups [17]. Synovial TNF-α, IL-1β, and MCP-1 levels were increased in the OA-control group compared with the Sham group. Specifically, TNF-α increased from Sham (25.0 ± 4.0) to OA-control (85.0 ± 10.0), IL-1β increased from Sham (18.0 ± 3.0) to OA-control (55.0 ± 8.0), and MCP-1 increased from Sham (120.0 ± 15.0) to OA-control (260.0 ± 25.0). These inflammatory markers were reduced after treatment, with the lowest values observed in the Vitamin D + Curcumin group: TNF-α (30.0 ± 5.0), IL-1β (22.0 ± 4.0), and MCP-1 (135.0 ± 16.0). In contrast, IL-10 was reduced in OA-control rats (45.0 ± 7.0) compared with Sham rats (80.0 ± 10.0) and increased most clearly in the Vitamin D + Curcumin group (88.0 ± 11.0).

## 4. Discussions

The effects of vitamin D, curcumin, and combined Vitamin D + Curcumin treatment on ACLT + MMx-induced knee OA were evaluated using functional assessments, cartilage histology, synovial Nrf2/HO-1 mRNA expression, serum oxidative stress markers, and synovial cytokine analysis. The present study showed that the ACLT + MMx procedure successfully induced knee OA-like pathological changes in rats and allowed reliable collection of synovial membrane tissue for further analysis [5,6,7]. As shown in Figure 2i, the operated knee joint was clearly exposed, excised with small femoral and tibial fragments, and opened from the femoral side to identify the patella, patellar ligament, and synovium-covered region. This confirmed that synovial tissue could be collected appropriately for molecular and cytokine evaluation [32]. Body weight increased gradually in all groups from week 0 to week 12, with no obvious weight loss (Figure 2ii), suggesting that both the surgical procedure and oral administration of vitamin D, curcumin, or their combination were generally well tolerated. Functional assessment confirmed that ACLT + MMx surgery caused joint discomfort and swelling in OA-control rats. The increased weight-bearing Δ force in the OA-control group indicated reduced loading on the operated limb, which may reflect pain-related functional impairment (Figure 3A). Similarly, the increased knee width suggested local swelling and inflammation after surgery (Figure 3B). Treatment with vitamin D or curcumin improved both functional parameters, while the combined treatment showed the strongest effect, indicating better recovery of limb use and reduced knee swelling.

Histological findings further supported the cartilage-protective effects of vitamin D and curcumin. The Sham group showed normal cartilage morphology, with a smooth articular surface and preserved matrix structure (Figure 4A). In contrast, the OA-control group showed typical degenerative changes, including surface irregularity, cartilage thinning, matrix disruption, and reduced chondrocyte organization (Figure 4B). These changes were reduced by vitamin D and curcumin treatment (Figure 4C,D), whereas the Vitamin D + Curcumin group showed the most evident cartilage preservation (Figure 4E) [23]. These histological observations were consistent with the OARSI scoring shown in Table 1. Quantitative cartilage analysis also confirmed the protective effect of the treatments. OA-control rats showed reduced cartilage thickness, altered cartilage width, and higher cartilage degradation and OARSI scores, indicating marked structural damage. Vitamin D and curcumin improved these parameters, and the combined treatment showed clear cartilage-protective effects. However, the cartilage degeneration and OARSI-related scores were close between the curcumin and Vitamin D + Curcumin groups, suggesting that the structural cartilage benefit of the combination was not uniformly greater than curcumin alone across all histological endpoints (Figure 5A–D).

The similarity between the curcumin and Vitamin D + Curcumin groups in some cartilage structural scores may indicate that curcumin produced a near-maximal histological protective effect under the present experimental conditions. In this context, adding vitamin D may not have further improved cartilage degeneration scoring to the same extent, possibly reflecting a ceiling effect for structural cartilage protection. However, the combination advantage appeared more evident in other outcome domains, including weight-bearing function, knee swelling, oxidative stress balance, synovial Nrf2/HO-1 mRNA expression, and inflammatory cytokine modulation. Therefore, the present findings suggest that the benefit of Vitamin D + Curcumin co-treatment may be domain-specific rather than uniformly superior across all measured endpoints. This interpretation should be considered exploratory, and future studies with larger sample sizes and domain-specific mechanistic analyses are needed to determine whether vitamin D mainly contributes to functional, redox, and inflammatory outcomes when combined with curcumin.

The molecular findings suggest that the observed cartilage protection may be associated with improved antioxidant signaling. Nrf2 and HO-1 mRNA expression were reduced in OA-control rats, indicating weakened antioxidant defense in synovial tissue (Figure 6A,B). Treatment with vitamin D or curcumin increased both markers, while the Vitamin D + Curcumin group showed the highest expression levels. However, because the present study evaluated Nrf2 and HO-1 only at the mRNA level, these findings should be interpreted as evidence of pathway involvement rather than direct proof of Nrf2/HO-1 pathway activation. Serum oxidative stress markers were consistent with this interpretation and suggested improved antioxidant balance after treatment [10,11,12]. OA-control rats showed increased MDA levels, indicating enhanced lipid peroxidation (Figure 7A). Elevated GSSG levels suggested disruption of glutathione redox balance (Figure 7B), while reduced CAT activity reflected weakened antioxidant capacity (Figure 7C). Vitamin D and curcumin improved these oxidative stress-related changes, with the combined treatment showing the greatest reduction in oxidative stress and the strongest improvement in antioxidant activity.

Synovial cytokine analysis demonstrated that ACLT + MMx-induced OA promoted a pro-inflammatory synovial response [14,15,16,17]. TNF-α and IL-1β levels were increased in OA-control rats, indicating activation of inflammatory pathways (Figure 8A,B). IL-10 was reduced, suggesting a weaker anti-inflammatory response (Figure 8C), while MCP-1 was elevated, indicating increased inflammatory cell recruitment (Figure 8D). Vitamin D and curcumin reversed these cytokine changes, and the combined treatment produced the strongest anti-inflammatory effect. Taken together, these findings suggest that vitamin D and curcumin, particularly in combination, protected against ACLT + MMx-induced knee OA by improving weight-bearing function, reducing knee swelling, preserving cartilage structure, increasing antioxidant-related Nrf2/HO-1 mRNA expression, lowering oxidative stress, and suppressing synovial inflammation. Overall, the combined treatment showed broader protective effects than either treatment alone in this preclinical OA model. The stronger effect observed in the Vitamin D + Curcumin group may be explained by complementary, rather than definitively synergistic, biological actions. Vitamin D may contribute to joint protection by supporting cartilage and subchondral bone homeostasis and by reducing inflammatory responses within the OA joint environment. Curcumin may provide additional protection through antioxidant and anti-inflammatory effects, including modulation of oxidative stress, inflammatory cytokine production, and antioxidant-related signaling. Based on the present findings, we hypothesize that the combined treatment may have produced broader protection by increasing antioxidant-related Nrf2/HO-1 mRNA expression, improving redox balance, reducing lipid peroxidation, and suppressing synovial inflammatory mediators. The reduction in MDA and GSSG, together with increased CAT activity and decreased TNF-α, IL-1β, and MCP-1 levels, supports the possibility that the two compounds may act additively on oxidative stress and inflammatory pathways. However, true synergism cannot be concluded from the present study because formal synergy analysis, NF-κB pathway assessment, glutathione synthesis or recycling markers, and pathway inhibition experiments were not performed. Therefore, this proposed combination advantage should be considered a mechanistic hypothesis that requires further validation.

The curcumin dose used in this study also requires careful interpretation in relation to clinical translation. In the present experiment, curcumin was administered at 100 mg/kg/day in rats. Using standard body-surface-area conversion, this dose corresponds to an approximate human-equivalent dose of 16.2 mg/kg/day, which is about 970 mg/day for a 60 kg adult and about 1130 mg/day for a 70 kg adult. This estimated range is broadly comparable with curcumin doses used in human supplementation studies; however, direct translation remains limited because conventional curcumin has poor aqueous solubility, rapid metabolism, and low oral bioavailability. Therefore, the present dose should not be interpreted as direct clinical dosing guidance. Future translational studies should consider pharmacokinetic evaluation and improved-bioavailability curcumin formulations, such as phospholipid complexes, micellar systems, piperine-containing formulations, or nanoparticle-based delivery systems, to determine whether clinically achievable exposure can reproduce the protective effects observed in this rat OA model. Several limitations should be considered when interpreting the findings of this study. First, the number of animals in each group was limited, which may affect the statistical strength of some comparisons. Second, only male rats were included in this study; therefore, the results may not fully reflect possible sex-related differences in OA development or treatment response. Third, although changes in Nrf2/HO-1 mRNA expression and inflammatory markers were examined, pathway inhibition experiments were not performed to directly confirm the involvement of this mechanism. Future studies using larger sample sizes, both male and female animals, and specific pathway inhibition approaches will help further validate these findings. Although Nrf2 and HO-1 mRNA expression was increased after treatment, protein-level validation, nuclear Nrf2 translocation analysis, and pathway inhibition experiments were not performed; therefore, the Nrf2/HO-1 finding remain associative rather than causal. Another limitation is that a formal sample size estimation or power calculation was not performed before the experiment. Although the group size was selected based on previous ACLT + MMx-induced osteoarthritis studies and prior experimental experience, future confirmatory studies should include an a priori power calculation based on clearly defined primary outcomes, such as OARSI score, cartilage thickness, knee width, or weight-bearing Δ force.

## 5. Conclusions

Our data suggest that vitamin D and curcumin provide protective effects against ACLT + MMx-induced knee osteoarthritis in rats, with the combined treatment showing broader overall benefits, particularly in functional, oxidative stress, molecular, and inflammatory outcomes, although some cartilage structural scores were similar to curcumin alone. The ACLT + MMx model successfully produced OA-like joint changes, including functional impairment, knee swelling, cartilage degeneration, oxidative stress, and synovial inflammation. Oral supplementation with vitamin D or curcumin improved weight-bearing function, reduced knee width, and helped preserve cartilage structure, while their combination produced greater improvement than either treatment alone. Histological and quantitative analyses confirmed that Vitamin D + Curcumin treatment reduced cartilage destruction, improved cartilage thickness, and lowered cartilage degradation and OARSI scores. At the molecular level, the combined treatment increased synovial Nrf2 and HO-1 mRNA expression, suggesting possible involvement of antioxidant-related signaling rather than definitive proof of direct pathway activation. This was supported by reduced serum MDA and GSSG levels and increased CAT activity, indicating improved oxidative stress balance. Additionally, treatment with vitamin D and curcumin suppressed synovial pro-inflammatory mediators, including TNF-α, IL-1β, and MCP-1, and promoted the anti-inflammatory cytokine IL-10. Overall, these results suggest that vitamin D and curcumin co-treatment may help slow OA progression in this preclinical ACLT + MMx rat model, possibly through complementary antioxidant and anti-inflammatory effects. However, whether this combination acts additively or synergistically requires further mechanistic and translational validation. However, further mechanistic studies and well-designed clinical investigations are required before this combination can be recommended for routine clinical management of OA patients.

## Figures and Tables

**Table 1:** Histology/OARSI-related scoring of OA knee joints. Data expressed as mean ± S.D. * *p* < 0.05, ** *p* < 0.01, and *** *p* < 0.001.

	Sham (*n* = 6)	OA-Control (Control) (*n* = 6)	Vitamin D (*n* = 6)	Curcumin (*n* = 6)	Vitamin D + Curcumin (*n* = 6)
1. Measured Cartilage matrix loss in millimeters	0 **	3.9 ± 0.6	2.9 ± 0.8	1.7 ± 0.2	1.19 ± 0.2
2. Measured Cartilage degeneration score	0 ***	10.3 ± 1.4	6.21 ± 2.3 *	3.1 ± 1.2 **	3.2 ± 0.6 **
3. Measured Total cartilage degeneration width in millimeters	0 ***	2.3 ± 0.4	1.82 ± 0.4	1.82 ± 0.2	1.1 ± 0.2 *
4. Measured Significant cartilage degeneration width in millimeters	0 ***	1.7 ± 0.23	0.76 ± 0.3 *	0.16 ± 0.1 ***	0.2 ± 0.1 ***
5. Measured Zonal depth ratio of lesions	0 **	2.1 ± 0.3	0.81 ± 0.4 *	0.51 ± 0.3 **	0.47 ± 0.2 **

## Data Availability

The original contributions presented in this study are included in the article. Further inquiries can be directed to the corresponding author.
